# Effects of development interventions on pastoral livelihoods in Turkana County, Kenya

**DOI:** 10.1186/s13570-021-00197-2

**Published:** 2021-11-24

**Authors:** Gregory Akall

**Affiliations:** Africa Office, United Nations Environment Programme, UN Avenue, New Office Block 2, Level 1, P.O. Box 30552-00100, Nairobi, Kenya

**Keywords:** Land use, Pastoralism, Irrigation, Donors

## Abstract

Turkana County has a long history of drought and development interventions and remains one of the poorest counties in Kenya. In Turkana, livelihoods are increasingly under threat because of climate change, conflict, and the changing land use and management. There are complex interactions between the multiple drivers of change in landscapes and livelihoods in the region. The question addressed here is: How have external development interventions contributed to the changing pastoralist livelihoods in Turkana? This study is specific to the lower part of the Turkwel River basin, particularly the Nanyee irrigated area in Turkwel, Loima sub-County of Turkana County. This article examines the external development interventions during the colonial, post-independence, and contemporary periods to reveal the ways that land use practices and livelihoods have changed across these periods. Land use practices are changing due to the growing human population, droughts, urbanization, and dispossession of grazing areas through state and donor-supported interventions. It is suggested in this article that the change from a system of customary, unrestricted grazing to one of enclosed pastures has threatened pastoral territories, as well as cultures and livelihoods over the past six decades. The new set of development interventions introduced by international and national actors have failed to support local livelihoods, instead joining the list of existing problems that undermine pastoralism, including drought, livestock diseases, and cattle rustling.

## Introduction

Kenya has a land area of 580,728 km^2^, of which 89% is classified as arid and semi-arid land (ASAL) or drylands. Nomadic or semi-nomadic pastoralism has long been the dominant form of land use in the dryland areas of northern Kenya (Adams 1992:46). Historically, developers have seen these areas as sites of famine, destitution and impoverishment, contributing little tax to State coffers (Lind et al. 2020; Catley et al. 2013:12). But pastoralists have for centuries been exposed to drought, conflict and famine, to which they have adapted strategic responses (Catley et al. 2013; Kratli 2013). People often depend on their own experience to understand changes in their environment (Castro et al. 2012:175). Pastoral systems make use of dryland environments by working *with* their characteristic variability rather than *against* it (Kratli 2013). Western et al. (2020, 7) contend that free-ranging movements give livestock access to resources over large regions.

In Turkana County, north-western Kenya, livestock keeping is the main economic activity for most people, supporting about 62% of the local population. Some 20% of the remaining population depends on agro-pastoralism, 12% on fishing, and 8% on casual labour (Turkana County Government 2013).

Turkana has been subjected to major droughts over the past 100 years. Many herders shifted to alternatives during the drought of 1979–1981 during which large numbers of livestock were lost to starvation and diseases (Little and Leslie 1999: 328–29). Consequently, herd owners moved into famine relief camps, food distribution sites, irrigation schemes, as well as small towns and trading centres in the pastoral areas (Lind et al. 2020; Opiyo et al. 2016; Little and Leslie 1999:335; Hogg 1986). The devastation of the drought was exacerbated by the risks of cattle raids, livestock diseases, and locust invasions similar to the most recent one in 2020. Historically, Turkana people have utilized a wide range of alternative strategies, depending on the severity and duration of drought. These tactics include mobility, rapid response to vegetation shifts, sharing information using social networks, and changing herd composition (Western et al. 2020; Catley et al. 2013; Little and Leslie 1999:329). Over the centuries, they have established a pattern of social organization, subsistence, and environmental manipulation, which has enabled them to survive in times of rapid change, scarcity, and uncertainty (Little and Leslie 1999:372–73). However, Turkana pastoralists have taken action to deal with the changing climate (Little and Leslie 1999:329).

### Pastoralism under threat

Twenty-twenty was one of the warmest years on record. Globally, more than 50 million people were reported directly affected by floods, droughts, or storms combined with the COVID-19 pandemic (UNEP 2021:6). Pastoral households worldwide are food insecure because of climate change. The semi-arid regions of Africa occupied by pastoralists are vulnerable to climatic and non-climatic risks (Dupar 2019:13). Climate change has deepened environmental uncertainties linked to an increasing severity and intensity of droughts, floods, livestock diseases, conflicts, small arms proliferation, locusts, and the COVID-19 pandemic (Simula et al. 2020; Mkutu et al. 2019; Lind 2018; Schilling et al. 2016, Schilling et al. 2015; Opiyo et al. 2015; Schilling et al. 2012). As historically recorded since the 19th Century, pastoralism has been under threat from these risks which have undermined livelihoods in the drylands. With global climate change increasing environmental instability, pastoralists are seen as among the groups most at risk (Opiyo et al. 2015; Flintan et al. 2013). Most of the drylands are rangelands, and stresses on land worsen existing livelihoods and food systems (Dupar 2019).

In 2010, the Constitution of Kenya was ratified giving rise to devolved units. The constitution created 47 counties and empowered them over key aspects of land administration, including the management of community land to improve focus on local priorities in land rights and land administration (Gargule and Lengoiboni 2020; Mkutu 2020). From 2016, the Community Land Act was enacted in Kenya, which saw recognition of customary land tenure (Cormack 2016). However, since the enactment of the Community Land Act 2016, the implementation of the Act has been slow and few people are aware of the rights that it endows on them (Mkutu 2020; Gargule and Lengoiboni 2020). According to Mkutu (2020), under the communal land regime, it is relatively easy to dispossess communities of their land without their participation.

Northern Kenya has featured in the country’s emphasis on infrastructure development for economic transformation (Lind et al. 2020; Lind 2018; Mosley and Watson 2016). These infrastructural projects include the Lamu Port-South Sudan-Ethiopia Transport (LAPSSET) Corridor, wind energy, oil and gas, water aquifers discovery, geothermal exploration, refugee camps, and military camps, among others (Mkutu 2020; Mkutu et al. 2019; Schilling et al. 2016; Opiyo et al. 2015; Flintan et al. 2013). All these developments required community-owned land and resources, thus already resulting in dispossession before the Community Land Act was ratified in 2016 (Mkutu 2020). In the counties of northern Kenya, elites are at the forefront of land privatization, involving fencing off prime grazing areas, for speculative reasons and rights to future compensation (Lind et al. 2020; Greiner 2016), especially from infrastructural development projects mentioned above. There is a strong discourse about private property ownership rights. As a result of this narrative, land speculation, land grabbing, and land-based disputes are high on the ground. Gargule (2019) explains that green energy grabbing of pastoral land is hidden in voluntary corporate social responsibility (CSR) through which dispossession is justified and legitimized. With these risks and impacts, mobile pastoralism is becoming increasingly constrained through land fragmentation because pastoralists lack access to good resources needed to manage uncertainty (Lind et al. 2020; Opiyo et al. 2015) which has reduced their resilience to extreme drought (Dupar 2019:11).

This article analyses the effects of development interventions on pastoralist livelihoods in the lower part of the Turkwel River basin in Turkana County, in response to socio-ecological changes and external development processes. This study applies a political ecology approach to frame “the complex relations between nature and society through a careful analysis of what one might call the forms of access and control over resources and their implications for environmental health and sustainable livelihoods” (Watts 2000: 257). The political ecology approach emphasizes the understanding of history, material conditions and processes, which feed into the policy-making process (Bryant and Bailey 1997). Those who are powerful and dominant protect themselves by retaining control over the poor, weak, and vulnerable (Chambers 1997). Policy elites make authoritative decisions in government by identifying problems, actualizing goals and important policy outcomes (Grindle and Thomas 1991). The article begins with a review of challenges facing pastoralism in Turkana and the transformations in the socio-economic environment. Secondly, it analyses the interaction of development interventions with communal-pool resources of land, crucial for sustaining pastoralism which is a natural resource-dependent livelihood. Finally, the article identifies discourses that reinforced a civilizing mission of settlement projects, irrigation schemes, provision of basic services, and social protection interventions more recently.

### Droughts, destitution, and development narratives

#### Drought and destitution

Droughts are a normal occurrence in Turkana. During the colonial period, severe droughts occurred in 1924, 1932, 1933, 1952, and 1960, necessitating famine relief (Oba 1992).

Destitute herders settled in towns, introducing an unprecedented urban lifestyle into the Turkana landscape. Former livestock keepers who survived the famines were recruited into temporary famine relief camps set up along Lake Rudolf (now Lake Turkana) in 1924, 1932–1933, 1960–1961, 1970, and 1980. Between 1937 and 1942, about 26 destitute families were maintained at Ferguson’s Gulf, where they survived on fishing. By 1959, there were 700 destitute families in relief camps (Hogg 1982). In 1960–1961, a severe drought pushed the number of impoverished pastoralists at famine camps to 30,000 at Lodwar and Lorugum famine relief camps. In 1972, about 42,000 people received food aid. In 1983–1984, some 80,000 out of 180,000 were supplied with relief food (Oba 1992; Hogg 1982). The district has suffered a series of 30 severe droughts between 1963 and 2019.^1^ These droughts decimated thousands of livestock, rendering many homesteads stockless. From the early 2000s up to 2014, the frequency of recurrent droughts increased, and during the 2011 drought, Turkana households lost an estimated 50 to 70% of their livestock (Bersaglio et al. 2015: 689).

#### Irrigation-based development

In the post-colonial period, there was a clear policy to encourage development and investment in the region. Kenya’s post-independence government and international agencies embarked on ambitious plans to introduce alternative livelihoods for the Turkana people. Major disasters, such as drought, raiding, insecurity, and diseases, reinforced the dominant narrative that destitution was caused by overstocking and desertification. This view encouraged destocking and resettlement of destitute herders (Catley et al. 2013:12). African pastoralism was viewed as archaic, resistant to change, and anti-modern (Cormack 2016:551), as well as stagnant, unproductive, and an ecologically damaging livelihood (Lind et al. 2020; Turner 2011:469). These perceptions influenced the development programmes and policies introduced by the State and donor agencies. The impact of the droughts prompted planners to promote an agenda for shifting from pastoralism to irrigated agriculture and fishing (Adams and Anderson 1988; Hogg 1987). Irrigated farming was believed to provide a livelihood, and settlement would allow government services to be provided, such as clean water, health facilities, and classroom education (Little and Leslie 1999: 337; Adams 1992). The planners saw the adoption of irrigation as a “privileged” technological response, while ignoring local experience (Moris and Thom 1990; Adams and Anderson 1988). Yet irrigation was not new in East Africa. In Kenya, Adams and Anderson (1988) mention that the simplest indigenous irrigation practice was recorded among the Turkana, who cultivated sorghum in the floodplains of the Kerio and Turkwel Rivers. Hogg (1987) argued that the settlement policy had little positive impact because it concentrated development resources in agriculture and fisheries, rather than livestock. While pastoralists had a history of interaction with settled farmers, they were not interested in switching from herding to cultivation. Limited cultivation was part of the complex of productive activities that they practised as herders, discussed later. They “took refuge” with irrigation farmers, and after accumulating sufficient livestock, they re-entered pastoral production (Anderson and Broch-Due 1999:247).

In Turkana, between 1966 and 1978, small-scale irrigation projects were started as a means to reduce dependency on food hand-outs, (Anderson and Broch-Due 1999:245) sedentarization, fishing, restocking, and land restoration. The schemes were designed to provide a “new livelihood” (sedentary life) for nomads affected by drought or raiding (Little and Leslie 1999; Anderson and Broch-Due 1999; Adams 1992; Hogg 1987). Irrigation was thought to have great potential in helping the locals to adapt to drought, build resilience, and improve food security. The intended role of pastoralists was to be operators (Catley et al. 2013:49). The period between 1990 and 2011 was characterized by humanitarian interventions due to the intense droughts experienced in Turkana (Bersaglio et al. 2015). The 2011 Horn of Africa drought stimulated afresh interest in finding a solution to the drought problem. The renovation of irrigation schemes that had fallen into disuse then followed. In 2013, UNESCO discovered two huge water aquifers in the Lotikipi plains and Napuu near Lodwar, estimated to hold enough water to supply the entire Kenya for the next 70 years (Avery 2014). This discovery triggered enthusiasm among developers, and in early 2016, the Turkana County Government launched a 650-ha drip irrigation scheme to utilize one of the aquifers just outside Lodwar town. In August 2016, the national Government, through the Kerio Valley Development Authority (KVDA), a parastatal with operations in the north Rift valley region, also launched a 150-ha solar-powered commercial pivot irrigation scheme to utilize the aquifer. All these external interventions have made little or no impact on the lives of local Turkana.

### Communal land and development projects

Customary pastoral land tenure in Kenya has been given renewed attention in light of a legal debate. Cormack (2016, 552) contends that the ratification of the 2010 Constitution gave legal provisions to protect communal land tenure, and recognized pastoralists as a “marginalized group”. The “Community Land” legislation is set to facilitate communal land holdings, as well as compensation for compulsory acquisition of any community land (RoK 2016). Before devolution in 2013, the County Councils held community land in trust for pastoralists. However, this responsibility has since been transferred to the devolved county governments. The Community Land Act (2016) allows the county government to hold in trust all unregistered community land and monies payable as compensation for compulsory acquisition of any unregistered land (RoK 2018). Overall, the protection of community land is essential for the sustainability of pastoralism, especially when it comes to development projects.

The effective management of commons is important for pastoralism and agro-pastoralism. Turkana people value their land as a source of livelihood as it supports livestock keeping, especially in the grazing reserves (*Amaire* or *ekitela*), riverine areas rich in herbaceous species which serve as dry season grazing areas, water sources and irrigated agriculture crop farming, especially along River Turkwel, and fishing along Lake Turkana. In Turkana, areas of woodland along seasonal rivers are vital to the survival of herds through the dry season.

Historically, riverine forests were managed through an indigenous system known as *ekwar*, which involves semi-private usufruct rights to the resources on designated parts of land, customarily associated with sections of riverbank. The *ekwar* is a parcel of riverine forest in which the owner and his close family members had the exclusive rights to collect non-timber forest products (NTFPs), such as building materials, firewood, edible fruits, and the pods from the *Acacia tortilis* tree, an important source of nutrition for livestock*.* Traditionally, customary land system of *ere * (or wet season grazing area and the permanent settlement where old and young stock may remain all year) and the *ekwar* are used to manage natural resources. The *ekwar* is family-owned and outsiders are required to seek access and user rights for dry season grazing and water sources (Barrow 1991; Barrow 1990:474). The woodlands on the banks of the rivers have often been the target of attempts to “develop” semi-arid regions (Adams 1992:49). However, since the 1960s, there have been conflicts between the *ekwar* system and the forestry and development interventions. The *ekwar* system has been eroded by alternative livelihood land-use practices, including irrigated agriculture, poultry farming, fodder production, and conservation (Stave et al. 2007; Haro and Oba 1993; Barrow 1990).

Similarly, from early 1990s to 2020, there has been pressure on communal land from drought, and degradation caused by population growth, including the setting up of the Kakuma refugee camp and Kalobeyei Integrated settlement, which house about 198,450 refugees (UNHCR 2020). There are also infrastructural development projects, such as oil fields, airport, planned resort city at Eliye Springs on the western shores of Lake Turkana, geothermal exploration, commercial irrigation, and rapid urbanization. In October 2020, Turkana County Government signed a Memorandum of Understanding with the Ministry of Defence to set up nine military camps for security purposes.^2^ For example, in Turkana, major and small towns are expanding and encroaching on dry season grazing areas. Further, the increasing land fragmentation and fencing of grazing reserves has blocked livestock migratory routes and denied local herders’ access to key common-pool resources.^3^

Community land politics is dominated by the national and County government officials, ruling elites and individuals with money and political connections, who were accused of promoting a narrative of private property rights.^4^ In addition, the County government has usurped the role of the traditional *Akiriket*, council of elders, who historically made decisions on community land. According to a civil society official, recent acquisition of community land for roads construction and oil exploration excluded traditional institutions.^5^ This respondent also claimed that the County government was reluctant to lose the custodian role of community land because once land is registered, the community takes control of its administration and management (Republic of Kenya 2018).

Overall, the problems of limited resources attributed to severe droughts, poverty, conflicts, population growth, communal land fragmentation, and dispossession are eroding the quality of land and undermining local Turkana pastoralist efforts to conserve common pool resources for the present and future users. So, this study helps to understand the effects of development interventions on pastoralist livelihoods in the past and present. This is in response to the new set of development projects and laws related to community land, including Land Act 2012, Community Land Act 2016, and Community Land Regulations Act 2018.

## Methods

Data used in this study comprises detailed oral histories, archival materials, focus group discussions (FGDs), participant observation, and key informant interviews, including representatives of national and county governments, NGOs, and local leaders. The data was obtained from 100 agro-pastoralist, pastoralist, and town-dwelling households from February to September 2014. Follow-up visits took place in February 2015, May–August 2019, and Sept–Oct 2020. The follow-up visits helped to observe any socio-economic changes among the participants in the study area. This provided an in-depth understanding of historical and social relations in Turkwel. During participant observation in 2014, I lived in Turkwel for 8 months to observe daily activities of households working in the irrigation schemes. Oral history was used to record major events, such as drought, famine, diseases, raids, and locust invasions. Archival materials and secondary literature were analysed to triangulate the histories.

A snowball approach was used to identify pastoralist and agro-pastoralist households, who had lived in the area for various lengths of time, many of them practising either livestock keeping or flood recession agriculture. These households also interacted with external interventions, including irrigated agriculture, water agriculture, digging canals and afforestation. Irrigation schemes were associated with destitute Turkana settlers (*amasikin* Turkana, who worked as labourers at the schemes and lived in the pauper’s camp (or permanent settlements) in Turkwel (locally known as Kaekorongole). Respondents were identified through referrals by those interviewed earlier, as well as support from a local research assistant. Ten focus group discussions (FGD) were conducted, each comprising six women and six men. They were conducted in the irrigated areas, trading centres, riverine forests, and villages. The FGD participants were purposively selected depending on their availability and willingness to participate in the study. FGDs were used to validate the information from all the interviews. Those interviewed were household heads (men and women).

**Fig. 1:**
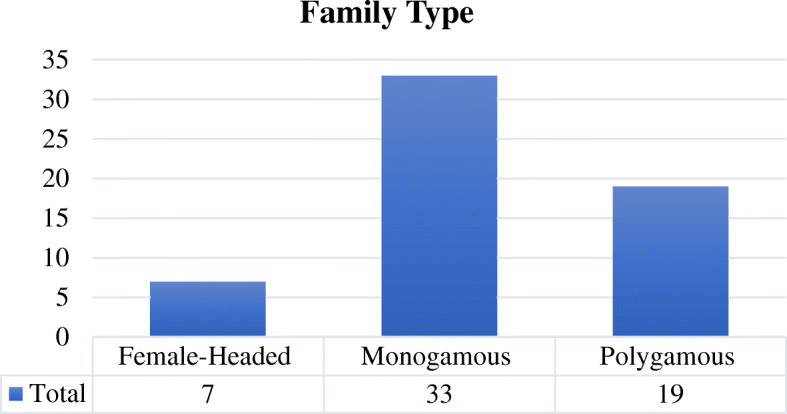
Family type

The sampled households were randomly divided into monogamous, polygamous, and female-headed categories. Figure 1 shows that there were 33 monogamous households, 19 polygamous ones, and a further seven headed by females. The oldest head of family was 85 years old, while the youngest was 29. The average age of household head was 59 years. The largest size of household was 40, including six to 25 children.

### Data analysis

The manual coding of qualitative data identified key themes in the discourse on external interventions. The data were transcribed from Ng’aTurkana and Kiswahili into English. Lists of topics, themes, and categories were developed through close, iterative readings of transcripts. The sections coded with related categories—such as “drought”, “flood cultivation”, and “irrigation”—were grouped together and critically analysed to compare the intended and realized effects of development interventions on pastoralist livelihoods.

### Study area

**Fig. 2:**
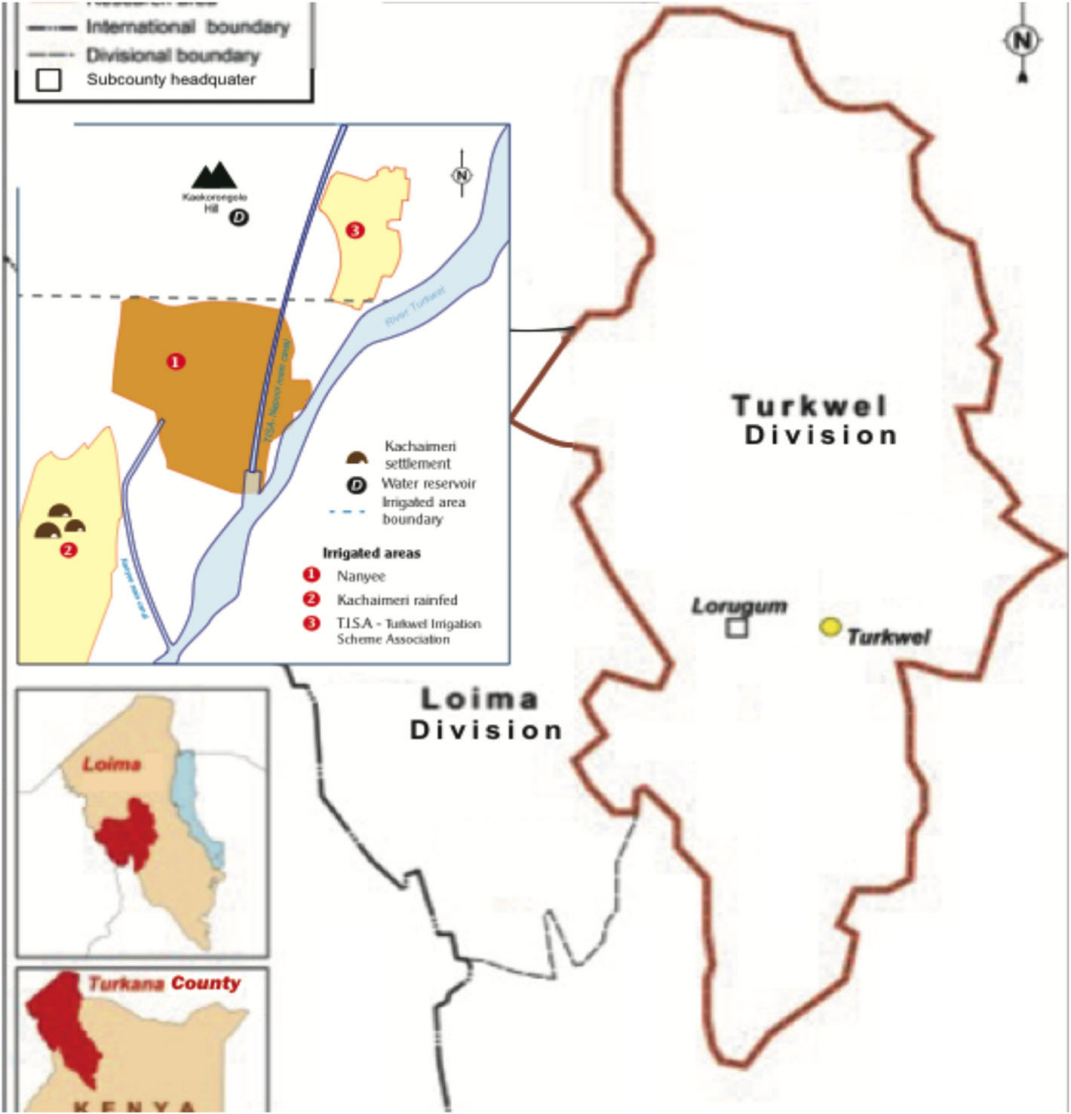
Location of the study area in Loima sub-County, Turkana

This study was conducted in Turkana County, north-west Kenya. More specifically, research was conducted in Turkwel Division of Loima sub-County, in the lower Turkwel River basin (Fig. 2). Turkwel Division has a population of 79,683. Loima sub-County has a population of 107,795 (KNBS 2019:124).

The dominant production system in Turkana over the past 200 years has been nomadic pastoralism, supplemented by flood cultivation along the Turkwel, Kerio and Tarach rivers. These production systems have endured since the early 1800s, with pastoralism serving as the widespread and culturally preferred option (Bersaglio et al. 2015; Lamphear 1992). Historically, the Turkana have utilized pasturelands that straddle the modern state territorial borders of Kenya, Uganda, Sudan, and Ethiopia. There were no boundaries separating Turkana and neighbouring pastoralist communities in these countries (Opiyo et al. 2016; Lokuruka and Lokuruka 2006; Collins 2006; McCabe 2004; Oba 1992; Lamphear 1992). However, in the twentieth century, colonial violence and the imposition of new forms of rule began to undermine the adaptability, mobility, and networks of Turkana, which had contributed to the viability of their livelihoods (Opiyo et al. 2015; Bersaglio et al. 2015: 689; Eriksen and Lind 2009).

Since 1966, the lower Turkwel River basin, a watershed spanning from Mt. Elgon to Lake Turkana, has been transformed by a large hydroelectric dam (Turkwel Gorge dam) and small-scale modern irrigation schemes. The basin is home to about 58,641 households—some 300,000 people—who practise both livestock and irrigated agriculture but the basin is bursting at the seams because of increasing stresses on the rangelands.

This site was chosen because Turkwel has a long history of flood cultivation, resettlement projects, and irrigation schemes, making it a useful location for inquiry into the historical and contemporary effects of development interventions on pastoralist livelihoods in Turkana.

In this article, I have studied one of the 19 territorial sections of the Turkana people, the *Ngmonia*. The *Ngmonia* who historically inhabited the Turkwel area (locally known as Kaekorongole) were engaged in agriculture and livestock keeping. Turkwel Location has an estimated population of 9,315 (KNBS 2019:124). It lies on the western bank of the Turkwel River, 35 km south-east of the county headquarters of Lodwar. This case study focuses on the Nanyee irrigated area, previously known as Kairuto. Since 1936, the local cultivators planted sorghum gardens using run-off water. But starting 1981, the Turkana Rehabilitation Project (TRP), an off-shoot of the famine relief efforts of 1981–1982, cleared 60 ha to set up Nanyee irrigated area. The European Economic Community (EEC), the present-day European Union, funded the project (Hogg 1982). The cleared field was converted into basin irrigation system; a surface irrigation method where ground water lifted from boreholes using a hand or motorized pump is applied, and later sub-divided into plots. The water was channelled using the force of gravity through a canal from the Turkwel River. The Nanyee irrigated area comprises 26 blocks, with a membership of 762 farmers in 2014.^6^

## Results and discussion

### Factors influencing pastoralist livelihoods’ change

The findings presented in this article discuss how pastoralist livelihoods have changed in Turkana’s Nanyee irrigated area, with particular attention to the influence of the Turkwel Irrigation Scheme Association (TISA), one of the earliest interventions to promote irrigation development. Moreover, I provide personal accounts of respondents on the Nanyee irrigated area, which was established in 1982 to address food shortage. The findings describe changes in the socio-ecological and economic landscapes brought about by external interventions, livelihood challenges, and emerging new lifestyles, as explained by respondents. Oral history evidence revolves around four households who have cultivated in or near Kaekorongole since the mid-1930s.

Based on interviews with elders of the *Ngmonia* territorial section (known locally as *Ngkwaamomwa*, people of the white sorghum), they have exploited flood cultivation in the Kachaimeri floodplains for well over one hundred years, fed by flash floods from the River Turkwel, Kangole and Konyipad streams. The local cultivators planted sorghum in flood-prone areas.^7^ More than 40 years after the introduction of sorghum in the Kachaimeri area, but before any serious involvement of colonial officials in irrigation, the *Ngkwaamomwa* were joined by impoverished settlers from other Turkana sub-groups who moved in voluntarily to the Turkwel riverine forest to live by gathering wild food and hunting.^8^ By 1936, *Ekaru a Eesomalit* (the year Somali traders arrived in Turkana), some of the earliest Turkana settlers in this area had acquired gardens through friendship, kinship, and marriage.^9^ They had also started cultivating sorghum gardens at Kachaimeri. The households increased from four to 14 before 1966, when modern irrigated agriculture and Turkana settlements were introduced in the area.^10^ Since the 1960s onwards, the Government of Kenya, the Food and Agriculture Organization (FAO), and church missions supported the resettlement of impoverished Turkana pastoralists from famine relief camps, to provide them with alternatives to pastoralism. In 1966, the Government and FAO moved some 1,000 destitute Turkana from famine relief camps and supported the establishment of the Turkwel Irrigation Scheme Association (TISA), previously known as Kaekorongole irrigation scheme, as a famine prevention measure (Akall 2020:158). The scheme took some 45 ha of communal land, mainly grazing areas of local herders. The scheme that was created benefitted 175 households (about 1,050 people). Another 100 ha was excised as settlements for the Turkana settlers, who became known colloquially as *Amasikin*, a term that combined the idea of “people of the scheme” together with the Kiswahili word for “poor” (*maskini*). FAO managed the scheme until 1978, when it was handed over to the Ministry of Agriculture (Akall 2020:159). This meant 145 ha of communal pool resources was converted to irrigated agriculture and settlement. This background has contextualized the evolution of irrigated agriculture activities in the area. The next section looks at Nanyee irrigated area; one of the new irrigation schemes, established in the early 1980s, two decades after the introduction of modern small-scale irrigation interventions in the area, which is the focus of this article. Nanyee is used as a case study to understand the effects of development interventions on pastoralist livelihoods in Turkwel area of Loima sub-County, past and present.

### Nanyee irrigated area

In 1982, NORAD, under the Turkana Rural Development Programme (TRDP), funded the scheme until 1990, when Norway and Kenya severed bilateral ties due to a diplomatic dispute (Akall 2020:159). It is worth noting that the destitute Turkana, who settled from 1980s until recently, do not identify with the *ekwar* system. This is because of the increasing informal privatization of land (with land ownership documentation being a letter of allotment or sale agreement) in small towns and across Turkana County. These newly-introduced irrigation schemes and settlements competed with the existing customary land use system, which was oriented toward livestock keeping. This has caused competition and changed power dynamics among livelihood groups in the area. For example, cultivating farmers denied pastoralists access to the dry season grazing areas and water points along the banks of the river. The competition between pastoralists and agro-pastoralists over traditional grazing areas increased conflicts along the river. The densely populated settlements also restricted access to grazing lands by pastoralists and also contributed to land degradation in the surrounding area (Adams 1992). State and non-state actors ignored pastoralism, even though it was the primary economic activity of the Turkana people (Hogg 1982). Irrigation interventions had limited local participation in their design and implementation. The irrigation programme ignored traditional flood cultivation methods and relied heavily on mechanization, as well as modern and expensive farm inputs.^11^

Following the 1980–1981 drought, Turkana Rehabilitation Project (TRP), which was initially funded by the Dutch government and European Union (formerly European Economic Community) registered destitute Turkana herders into a famine relief camp to facilitate food distribution. There was a shift in thinking from relief camps to livelihood interventions. TRP heralded cultivation as the path to sustained economic development (Hogg 1982). As a solution to the food problem, in 1982, TRP under the Turkana Rural Development Programme (TRDP) funded by the Norwegian government, recruited women from the destitute Turkana into food-for-work programmes to clear land for setting up sorghum gardens at Nanyee. They cleared vegetation from 60 ha of land, which was sub-divided into plots. TRP used farmers to build a canal dug from the Turkwel River, after which the basin irrigation system was introduced.^12^

A group of resident *Ngmonia* Turkana, who relied on seasonal cultivation at the 100 ha Kachaimeri, and the new destitute Turkana *Amasikin*, benefitted from these plots; each owning between four and 23 basins (0.08–0.46 ha). A basin is a surface irrigation method where water is applied to a nearly level field (in both directions and each unit (2.5-3m square) enclosed by dykes) through water raising by a bucket or by motorised pumps (Adams and Carter 1987) .^13^ According to Hogg (1982), most farmers received small farming tools, such as hoes, machetes, and shovels from TRP.

The Nanyee irrigated area received external support from TRP until 1990, when NORAD withdrew funding after Norway and Kenya severed bilateral ties. From 1990 onwards, Nanyee remained a farmer-led success story because of the flexible farming practices that combined both crop cultivation and livestock keeping. In 2000, farmers constructed their own secondary canal that serves up to 200 of them. In 2003, FAO installed canal structures, such as intakes and checkboxes. In 2005, Nanyee was registered as a water users association (WUA) under the District Gender and Social Services unit.^14^ This was in response to the reforms brought about by the 2002 Water Act, which liberalized the water sector, particularly the management of the river basin.

This meant that the scheme had to be managed through a formal administrative structure, as per WUAs’ rules and regulations. The scheme’s secretary mentioned:

The registration of Nanyee as a WUA enabled us to access external support as a communal group. For example, the Catholic Diocese of Lodwar introduced the seed revolving fund for Ksh.120, 000 ($1,200), and a further Ksh.370, 000 ($3,700) for nursery management.^15^

Additional support came from the Arid Lands Resource Management Project (ALRMP)—present-day National Drought Management Authority (NDMA)—which supplied farm tools and used farmers to build a tertiary canal in 2012 through a food-for-work programme supported by World Vision Kenya.^16^

Every 2 years, WUA members elect a governing committee. The committee handles issues like water use and management through block or tertiary system, mobilizes farmers to desilt the canal, and resolves conflicts between farmers and pastoralists or among the farmers themselves.^17^ In 2017, World Food Programme (WFP) helped to concrete-line the canal intake and fenced 400 m of the scheme, leaving a large part unfenced.^18^ Today, Nanyee irrigated area is facing the problems of wildlife (e.g. warthogs, monkeys, and birds), livestock invasions, thieves, water scarcity, pests, salinity, shrinking cropland, uncontrollably growth of *Prosopis*, and growing population.^19^

#### Livelihood strategies and household types

Based on income sources and structure, the study results showed that 46 households practised irrigated agriculture and 37 kept livestock, while 34 engaged in both irrigated agriculture and livestock keeping. Based on gender, 18 men aged between 36 and 82 years were involved in irrigated agriculture. The mean age among these was 61.

**Fig. 3:**
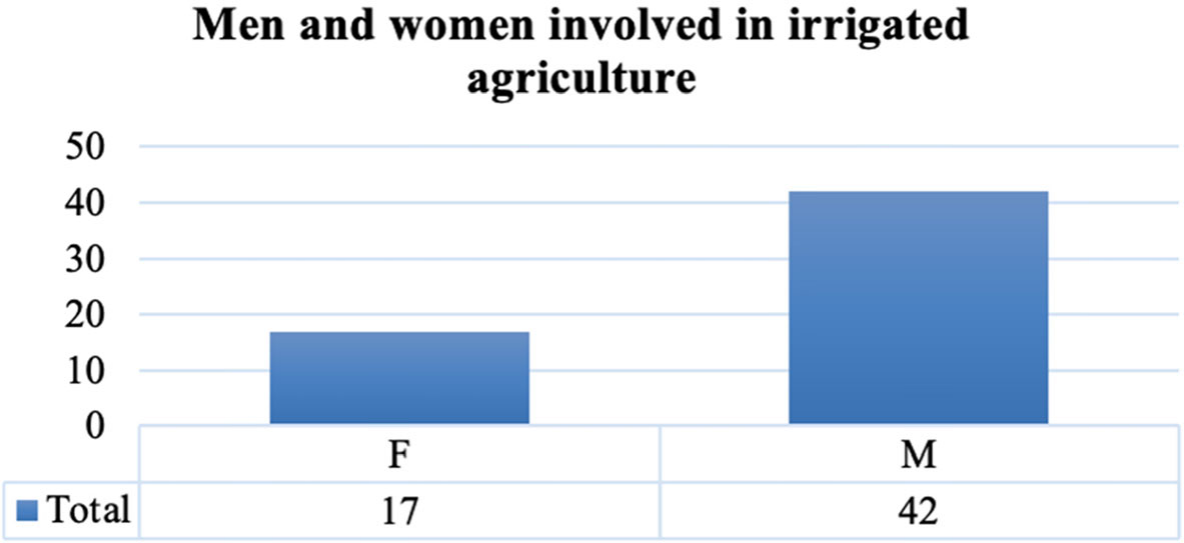
Men and women involved in irrigated agriculture

Some 11 women, among them two young women aged 34 and 35 years old, were involved in irrigated agriculture. The age range in this category was between 34 and 66 years, with a mean of 53 years. Overall, 42 men and 17 women practised irrigated agriculture (Fig. 3). The number of men was higher than that of women because culture demands that men inherit fields from their fathers. A farmer said: “My sons would inherit the fields from me because my daughters are married off and start their own families.” Notably, many women interviewees owning fields were widows, while some reported inheriting fields from their parents. There was a clear indication that inheritance of cropland by daughters was high in daughter-only households, or those headed by women. This meant that compared to men, women and girls have less access to own land. Their ability to benefit from irrigation interventions is thus diminished. As a result, some engaged in high-risk adaptation strategies, such as charcoal production and beer brewing beer, as discussed later.

#### Woody vegetation encroachment

Historically, intermittent floods recharged groundwater reservoirs, which sustained a mixed woody vegetation dominated by *Acacia tortilis*, *Hyphaene compressa*, *Salvadora persica*, *Ziziphus mauritania*, and *Cordia sinensi* (Adams 1989). However, in 1984, NORAD, through TRP, introduced *Prosopis juliflora*, originally from tropical America (Maundu et al. 2009), known locally as *etirae*. This plant was meant to reclaim the degraded land.

According to a forestry adviser involved in developments at the time, the Turkana were knowledgeable about their environment, but external interventions ignored their knowledge and capabilities.^20^ However, *Prosopis* has displaced indigenous woodland vegetation and grows uncontrollably in irrigated areas. This affects the activities of cultivators and pastoralists, who depend on the riverine forest for grazing, wild foods, and medicine. A frustrated cultivator at Nanyee, 73, explained:

*Etirae*, *Prosopis* has covered fallow fields because of its rapid spread. We now cultivate 20 out of 60 hectares cleared in the past.^21^

The alien invasive species has negatively impacted on local livelihoods. One example is a respondent age 83 and a pastoralist, who shared his frustration: “I lost 40 out of 50 goats to *Prosopis* after the pods blocked their rumen and damaged their teeth.”^22^

The result showed that the highest number of livestock (small stock, mainly goats) owned by a household was 800 goats in 1966 and has reduced to 10 goats in 2019. The average number of livestock owned by a household was 109. Households mentioned owning between 10 and 90 goats. The indigenous vegetation useful during dry season and lean periods has been displaced owing to the invasiveness of *Prosopis*, thus undermining livelihoods. Despite these disadvantages, local Turkana women are using *Prosopis* thickets as hideouts while brewing illicit liquor. They also use the wood for firewood, poles for building, and twigs for fencing homes and farms.^23^

#### Irrigation-induced population pressures

Resettlement schemes may have aimed at improving livelihoods of impoverished Turkana, but the narratives and experiences of members of the study population indicate that the opposite was true. The resettlement took place through several processes. First, the settling of destitute Turkana increased the population of Kaekorongole from 14 households in 1965 to 175 households in 1966. This constituted about 1000 people, all living in three villages. However, Hogg (1982) reported that by the early 1980s, there was over 3000 permanent residents in a landmass of 8 km^2^. At present, the Turkwel population is estimated at 9315 (or 2005 households) (KNBS 2019: 124) covering 12 permanent villages.

Secondly, the clearing of land to set up irrigated areas dispossessed agro-pastoralists and pastoralists of their seasonal rain-fed sorghum cultivation, dry season grazing land, and water sources along the banks of the Turkwel River. Oral history interviews showed that the 14 households mentioned earlier cultivated 100 ha from the Kachaimeri floodplains, Kairuto (present-day Nanyee) up to the foot of the Kaekorongole hill, for seasonal rain-fed sorghum cultivation.^24^

The seasonal sorghum gardens were productive and high yielding. An elderly farmer, who cultivated Kachaimeri floodplain garden, remembered:

I harvested up to 60 bags of traditional white sorghum from my seasonal garden at Kachaimeri. However, from 1966 to 1982, we lost over 100 hectares to the Kaekorongole water reservoir, which diverted water to the Kaekorongole irrigation scheme.^25^

To address this water shortage, in 1970, FAO constructed an embankment by diverting flash floods water to supply the sorghum gardens. But the water problem persisted because of drought. The same respondent further said:

In 1985, *Ekaru a namoco*, year Namoco was killed by suspected Pokot bandits, the Konyipad seasonal stream changed its course to the Turkwel River, denying the sorghum gardens run-off water.

This meant locals had to cultivate only during the rainy season. In 1982, a group that cultivated seasonal gardens at Kachaimeri moved to Nanyee as a risk spreading strategy. Thirdly, the clearing of land destroyed the indigenous woody vegetation that traps run-off water to prevent soil erosion. Furthermore, the enclosed irrigated areas blocked livestock migratory routes. The fencing changed the social dynamics among livelihood groups by limiting access to resources and precipitating tensions. For example, respondents mentioned that pastoralists trek up to 30 km in search of pasture. This has also caused conflicts between farmers and herders.^26^

Today, there are nine irrigated areas in Turkwel location, covering 1000 ha of pastoral communal land.^27^ Overall, this article suggests that these livelihood challenges have forced the study population to diversify to compensate for livelihood changes caused by resettlement and irrigation schemes as discussed below.

#### Shrinking fields and decline in agricultural production

Findings indicate that livelihoods in Turkwel are on a new trajectory. Agricultural production has declined significantly. This is attributed to factors like shrinking cropland, salinity, and water scarcity. The results showed that 46 households involved in irrigated agriculture owned plots of between two and 40 basins (the average size of a basin is 20 m by 10m). The average size of a household’s plot was eight basins. Majority of interviewees attributed reduced cropland to the invasive *Prosopis juliflora.*. This is evidenced by changes in the physical landscape, especially the increasing expanse of abandoned fields.

Results showed that households experienced low yields, with cultivators harvesting between 20 and 1500 kg (30 bags of sorghum/maize) from their fields.^28^ The average yield per basin was 36 kg. This is a substantial decline relative to the past. A respondent, 82,^29^ remembers good old days: “Some 40 years ago, I harvested 4,500kg to 5,400kg (50 to 60 bags of sorghum) from this seasonal rain-fed garden.”^30^

The various changes and adversities mentioned above have forced households to make tough choices regarding their livelihoods. Activities vary greatly across different categories of households as discussed in the next section.

### Alternative livelihoods and reduced options

The historical livelihood system of the Turkana provided ample opportunities for diversification to adapt to climate variability and other shocks. While much of diversification took place within pastoral production, such as mobility, herd diversification, and opportunistic planting, several authors have also described alternative livelihoods (Omolo and Mafongoya 2019; Schilling et al. 2016; Opiyo et al. 2015). For example, destitute Turkana in Turkwel engaged in alternative livelihoods like trading in food relief, beer brewing, petty trade, and selling charcoal and fuel wood. Notably, Hogg (1982) described that Turkana herders have historically treated famine relief food as a supplement to pastoral production.

**Fig. 4:**
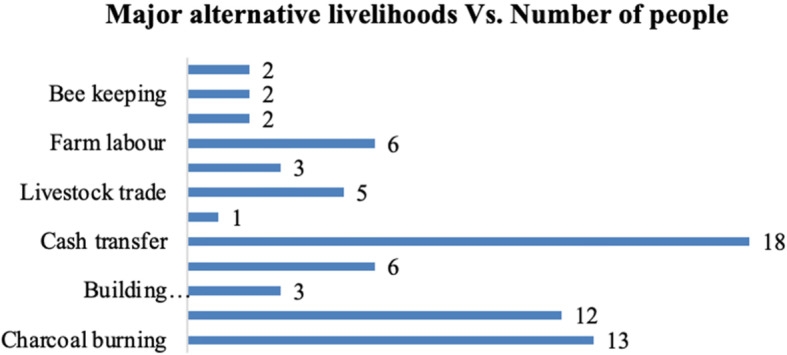
Major alternative livelihoods vs. number of people

The irrigation schemes forced them into a limited set of alternative livelihoods with lower productivity. This study suggests continuity of these alternative livelihoods in Turkwel. In the households interviewed, women often combined a variety of tasks-for-cash activities, such as charcoal burning, beer brewing, basketry, building materials, horticultural production, and beekeeping to compensate for the low crop yields and supplement household income. The irrigation schemes reduced the choices available to pastoralists. As illustrated in the specific examples of alternative livelihoods below and Figure 4, local Turkana continued to adapt through supplementary livelihood activities.

Figure 4 indicates that 47 households of sample population were dependent on alternative livelihood sources, such as cash transfer, charcoal burning, basketry, livestock trade, building materials, beer brewing, horticultural production, and beekeeping. An average of seven households of those interviewed depended on alternative livelihoods.

#### Cash transfer payouts

Famine relief has featured prominently in humanitarian responses in Turkana. However, food relief supplies took place until 2011, after which there was a lull following the introduction of other social protection interventions like cash transfer. Food relief distribution was cut off at some point. A respondent recalled: “We received food relief ration in 2010.”^31^ The decrease in food relief took place in parallel to an increase in a social safety net system of cash payouts under the Hunger Safety Net Programme (HSNP), known locally as *Lopetun* (in abundance).^32^ However, not everyone who was registered benefited from the cash transfer. A disappointed resident shared his frustration: “They (NGOs and Equity) came and registered us for this programme but majority of the peopled missed out. Our names were missing in the register, and some who received ATM cards are still waiting for cash two years later.”^33^ Despite the challenges, some are laughing to the bank. Cash transfer payouts form the highest share of livelihood income in the study site, and sometimes, the only source of income for households. Majority of households (20%) were dependent on cash transfer that makes up half of their income compared to other livelihood options. This contribution has become increasingly important, as other income sources decline. The average age of cash transfer beneficiaries was 61 years, with the oldest aged 82 years. Many elderly interviewees stressed being unable to continue with physical work like tilling land.

#### Charcoal production

The non-timber forest products (NTFPs) contributed to the second share of livelihood income among the study population. The results showed a mean income share of 13%. The results indicate interactions between households’ ownership of *ekwar* and engagement in charcoal burning. Other households engaging in charcoal burning relied on deadwood gifted by friends or bought from *ekwar* owners. Hogg (1982) wrote that the settling of destitute herders and clearing of bush for sorghum gardens had long-term effects on local environmental conditions, especially deforestation. As irrigation income diminishes, cultivators are exploiting the woodland vegetation to get alternative forms of livelihood. Majority of those involved in charcoal burning were elderly men, attributed to their ownership of *ekwar,* and bond friends who owned *ekwar* with a mixed wood vegetation of *Acacia tortilis* trees popular with charcoal production. For instance, results showed that a household burns a deadwood tree that produces about 25 bags (90 kg) of charcoal. However, due to ageing, some people are unable to produce enough charcoal. A respondent mentioned: “I burn between two and four bags of charcoal in two weeks. I sell a bag of charcoal for Ksh.300 ($3), mainly to passing middlemen in Turkwel.”^34^ The sample population used the proceeds from charcoal production to buy food, and pay school fees and hospital bills. The growing urban population in Turkwel and the nearby towns contributed to the demand for fuel wood energy. The results showed that other non-timber forest products, including basketry, formed the third source of income at 12%. Respondents engaged in basketry used *Hyphaene compressa* (*eengol*), native vegetation. The results indicate the overwhelming use of *Hyphaene compressa* for basketry, roofing materials, poles and edible nuts, and the invasive *Prosopis* would deplete it.

#### Beekeeping

In the past, local Turkana harvested honey for subsistence from the trunks of *Acacia tortilis* trees on the banks of River Turkwel. As irrigation scheme yields diminish, some households are diversifying into beekeeping to supply honey to growing urban markets. Beekeeping initiatives were started with members’ own capital and some external support. A group official said: “We use conventional beehives and protective equipment supplied by donor agencies and bought by the groups. We keep beehives in one of our member’s *ekwar* along the riverine forest.”^35^ The groups have established a strong connection with other producers, brokers, and consumers to achieve high volumes and high value. The honey value chain is sustained through maintaining a network of beekeepers along the Turkwel River.

The same official added:

We buy honey from other producers for Ksh.1, 400 ($14) for five litres, making a profit of Ksh.100 ($1). Our group has a membership of 12. We produce a minimum of 100l per season, making Ksh.30, 000 (USD 300) in Turkwel, and Ksh.50, 000 (USD 500) whenever we sell in Lodwar. By 2018, the group had savings of about Ksh.500, 000 (USD 5,000) from honey production.

The 2017 drought and wildfire outbreak that destroyed the woody vegetation affected the groups’ beekeeping income.

#### Horticultural production

Since the 1960s until 1990s, the Government and donor agencies promoted cultivation of market-value crops, such as dates, cotton, fruits, vegetables, and fodder in the small-scale irrigation schemes along River Turkwel. However, after the withdrawal of external support in the 1990s, the cultivation stopped. Only those who worked as labourers or had acquired horticultural farming skills continued cultivating. Over time, as staple food crop yields diminished, some households have diversified into horticultural farming and also invested in motorized water pumps costing between Ksh. 22,000 ($220) and Ksh. 50,000 ($500).^36^ A local irrigation schemes coordinator mentioned that by 2019, about 30 irrigators in Turkwel Location, which has 9 irrigated areas, including Nanyee (for example, in Nanyee, which is the focus of this study, only one farmer owned a motorised water pump), owned motorized water pumps, up from only two in 2014.^37^

To compensate for water scarcity in the irrigated areas, one farmer and a local church pastor, who owns a motorized water pump, said:

I bought a second-hand motorised water pump at Ksh.20, 000($200) to draw water from the *Amokololo* lagoon for vegetable production. I grow vegetables like kale (*sukuma wiki* in Kiswahili), tomatoes, onions, and cowpeas for sale in Lodwar. I earn about Ksh.30, 000 ($300) per month from vegetables.^38^

Households are using proceeds from vegetable sales to buy livestock, fuel for the water pump, and seeds.

Some people have found success with cultivation in the irrigation schemes, especially those with adequate financial capital. With enough initial capital, profits are guaranteed. However, start-up capital and social connections are likely to be unavailable to those who are the most vulnerable.

Unlike the cultivators mentioned with connections, and the local elite involved in horticultural farming, the majority of elderly farmers interviewed mentioned decried lack of money to buy motorized water pumps to boost their cultivation. Instead, these respondents conserve the riverine forest ecosystem using the *ekwar* system for long-term sustainable use.

#### Beer brewing

In the early 1980s, Hogg (1982) wrote that women among the resettled destitute Turkana, who had access to alternative food to famine relief maize, used the maize they received to brew beer. They used the profits to acquire livestock. This study found a continuity of women involvement in beer brewing. During fieldwork, a group of women brewing hard alcohol (*chang’aa*) in the thorny thicket of *Prosopis juiflora* fled after mistaking us for policemen. This showed that the brewers operated under constant fear of the law. In a focus group discussion with 20 women, it became clear that the reason for engaging in illicit brewing was to compensate for low crop yields. It was also a means of gaining start-up capital for possible expansion into other livelihoods. A brewer said: “I make up to Ksh.2, 000 (USD 20) in a day from the bootleg.”^39^

#### Multiple alternative forms of income

As shown in Figure 4, some households engaged in more than one alternative form of income, including charcoal production, farm labour, making *makuti* (roofing material) and mats, and selling building poles and firewood, to pool income. Households are using the earnings to buy livestock, clothes, food, and pay school fees. In summary, these results indicate how external interventions have influenced changes in pastoralist livelihoods in the study site. Interactions ranged between choice of household income strategies and local environmental conditions.

Oral history accounts showed that the study population has practised irrigated agriculture well over 100 years, alongside pastoralism before their interaction with external interventions. This article suggested that external development affects local livelihoods in both expected and unanticipated ways. Despite the underperformance of external interventions, local households have persisted in practising irrigated agriculture using some aspects of flood cultivation to sustain their livelihoods.

Similarly, sedentarization through settlements, irrigation schemes, the alien invasive species *Prosopis juliflora*, and urbanization are having effects on cultivators and pastoralist communities dependent on the riverine forest. The households are increasingly commodifying and engaged in alternative livelihoods to compensate for low crop yields and loss of pastoral livelihoods. Turkana land is under threat from a growing human population, displacements, and dispossession of grazing areas. These new challenges are putting more pressure on land, while the local Turkana remain impoverished.

## Conclusions

Rangeland fragmentation, involving conversion of grazing areas into irrigation schemes and permanent settlements or small-towns growth is constraining mobile pastoralism. The acquisition of land in Turkwel for irrigation development and resettlement of impoverished Turkana was undertaken without regard for the many hundreds of Turkana people and livestock that use the Turkwel riverine forest. Pastoralists lack access to key communal-pool resources, which they have used to cope with climate variability historically. This has resulted in disruption of livelihoods and straining of household incomes, with negative consequences for people’s ability to cope with future shocks.

Development interventions have contributed to inequalities along the lines of gender, education level, and socio-economic status, resulting in unequal access to state- and non-state-supported “alternative livelihoods” opportunities. As shown in this article, those with power, capital, and connections (i.e. men, mostly the elderly) are the ones who can invest in motorized water pumps and private land ownership, and it is minority that enjoys a disproportionate share of the benefits.

The Constitution of Kenya 2010 recognizes the equal rights of women and men to inherit land. Art. 60 (1) (f) is a commitment to eliminating gender discrimination in law, customs, and practices related to land and property (Republic of Kenya 2010). However, in Turkana and Nanyee in particular, women and girls are largely unable to inherit farmland. This means women and girls are disadvantaged and excluded from participating in state- and non-state-funded development projects, such as irrigated agriculture. They are instead involved in high-risk alternative livelihoods strategies, such as charcoal burning and beer brewing, as a means of acquiring capital for possible expansion into other livelihoods.

The problems of human population growth, demand for land, and water for irrigation and livestock are depleting natural resources and threatening pastoral livelihoods. In addition, the mushrooming of small towns is contributing to over-exploitation of natural resources, especially riverine forests that are cut for wood fuel to supply the growing urban populations. It is evident from this article that the sampled population are already experiencing low livestock outputs and low crop yields. Nanyee residents increasingly engaged in alternative livelihoods to compensate for low crop yields and loss of pastoral livelihoods. This article has also highlighted that households, which were traditionally dependent on either irrigated agriculture or livestock keeping, combined both livelihood systems to diversify their livelihoods, along with the supplementation of charcoal production, beekeeping, horticultural production, and beer brewing.

Poor households in Turkwel are becoming increasingly dependent on cash income, including that provided through cash transfer programmes as well as earnings from charcoal burning, basketry, livestock trade, building materials, horticultural farming, beekeeping, and beer brewing. These changes can be attributed to the transformations of the local landscapes and livelihoods, such as a decline in agricultural production, woody vegetation encroachment, and loss of grazing land to irrigation schemes and settlements. In turn, competition for limited natural resources (e.g. land, water, development interventions, like irrigation) is likely to cause future pauperization and conflicts among pastoralist, agro-pastoralist, and urban residents (interview with a conflict and peacebuilding expert in May 2019).

This study recommends the implementation of the Community Land Act 2016 to enable local communities to register their communal land, which allows spatial and temporal access to larger rangelands and protect emerging dispossession of rangelands by elites and politically connected individuals in sub-national governments. It also recommends that development planning should appreciate the potential of local knowledge and adaptive capacities to address socio-ecological changes and emerging vulnerabilities. According to UNEP (2021:46), urgent action is needed to promote nature-based solutions for adaptation, which can be used to restore, build, and enhance ecosystem services to help marginalize groups like pastoralists, to adapt to climate change and enhance their resilience, assets (livestock), and society.

## Data Availability

All data generated or analysed during this study are included in this published article and available from the corresponding author on reasonable request.

## Abbreviations

ALRMP: Arid Lands Resource Management Project
CSR: Corporate social responsibility
EEC: European Economic Community
EU: European Union
FAO: Food and Agriculture Organization for the United Nations
FGD: Focus group discussions
GoK: Government of Kenya
HSNP: Hunger Safety Net Programme
KNBS: Kenya National Bureau of Statistics
KVDA: Kerio Valley Development Authority
NDMA: National Drought Management Authority
NGO: Non-Governmental organizations
NORAD: Norwegian Agency for Development Cooperation
NTFPs: Non-timber forest products
RoK: Republic of Kenya
SEI: Stockholm Environment Institute
TISA: Turkwel Irrigation Scheme Association
TRDP: Turkana Rural Development Programme
TRP: Turkana Rehabilitation Project
UNDP: United Nations Development Programme
UNEP: United Nations Environment Programme
UNESCO: United Nations Educational, Scientific and Cultural Organization
UNHCR: United Nations Commissioner for Refugees
WFP: World Food Programme
WUA: Water users association

## Footnotes

1 This data is derived from Republic of Kenya (2013:46), Republic of Kenya (2012), Fratkin et al. (2011: 2), SEI (2009:17), Oba (1992), and Brainard (1986).

2 Interview with Turkana County Attorney on 5 October 2020.

3 Interview with a group of herders in Kanamkemer in September 2020.

4 Interview with a Peace & Justice official at the Catholic Diocese of Lodwar on 6 October 2020.

5 Interview with a Lodwar-based civil society land activist in September 2020.

6 Interview with the chairman of Nanyee irrigated area in June 2014.

7 Oral history account indicates that the Turkana acquired traditional white sorghum from the *Ngmarille* (Dassanech) agro-pastoralist of south Ethiopia in 1890.

8 Interview with 82-year-old elder at Nanyee in June 2014

9 FGD with residents of Kachaimeri in August 2019.

10 Interview with chairman of Nanyee irrigation (whose father migrated into Kachaimeri in the 1930s) in August 2019.

11 Interview with a former programme manager in Sept 2013.

12 A group discussion with farmers at Nanyee irrigated area in June 2014.

13 The average basin is 20 m by 10 m.

14 Interview with coordinator of the Turkwel irrigation schemes in March 2014.

15 Interview with Nanyee secretary in August 2019.

16 Interview with Nanyee irrigation chairman in July 2014.

17 Interview with coordinator of the Turkwel irrigation schemes in March 2014.

18 Interview with secretary of Nanyee irrigation scheme in August 2019.

19 A group discussion with farmers at Nanyee in June 2014.

20 Interview with a former forestry advisor in September 2013.

21 Interview with a farmer at Nanyee in June 2014.

22 Interview with a herder at TISA in March 2014.

23 A focus group discussion with Turkana women in June 2014.

24 FGD with Nanyee residents in May 2019

25 Interview with 82-year-old elderly farmer in June 2014.

26 Focus group discussion with residents of Nanyee in August 2019.

27 Interview with an agricultural officer in Turkwel in February 2015.

28 The average bag is 50 kg.

29 Interview with a farmer (who cultivated the Kachaimeri floodplain gardens and now owns a plot at Nanyee) in July 2019.

30 The average bag is 90 kg.

31 Interview with a former official of TISA in March 2014.

32 During its pilot phase (2007–2012), those interviewed claimed that everyone was registered regardless of their socio-economic status; thus, the programme became known locally as *Lopetun*.

33 Interview with a cash transfer hopeful in August 2019.

34 Interview with a farmer at Nanyee, who engages in charcoal production to compensate for low crop yield, in July 2019.

35 Interview with the secretary of the beekeeping group at Nanyee in August 2019.

36 A focus group discussion at Nanyee irrigated area in August 2019.

37 Interview with the Turkwel irrigation schemes coordinator in August 2019.

38 Interview with a farmer at Nanyee irrigated area in August 2019.

39 Interview with a woman brewer in the bushy *Prosopis* thicket at Napool in July 2014.
